# QTL Mapping of Agronomic and Physiological Traits at the Seedling and Maturity Stages under Different Nitrogen Treatments in Barley

**DOI:** 10.3390/ijms24108736

**Published:** 2023-05-13

**Authors:** Zhaoyong Zeng, Shiyun Song, Jian Ma, Deyi Hu, Yinggang Xu, Yao Hou, Chengjun He, Xiaoyan Tang, Ting Lan, Jian Zeng, Xuesong Gao, Guangdeng Chen

**Affiliations:** 1 Triticeae Research Institute, Sichuan Agricultural University, Chengdu 611130, China; 2 College of Resources, Sichuan Agricultural University, Chengdu 611130, China13982794374@163.com (C.H.);

**Keywords:** nitrogen stress, barley, traits, quantitative trait locus, seedling and maturity stages

## Abstract

Nitrogen (N) stress seriously constrains barley (*Hordeum vulgare* L.) production globally by influencing its growth and development. In this study, we used a recombinant inbred line (RIL) population of 121 crosses between the variety Baudin and the wild barley accession CN4027 to detect QTL for 27 traits at the seedling stage in hydroponic culture trials and 12 traits at the maturity stage in field trials both under two N treatments, aiming to uncover favorable alleles for N tolerance in wild barley. In total, eight stable QTL and seven QTL clusters were detected. Among them, the stable QTL *Qtgw.sau-2H* located in a 0.46 cM interval on the chromosome arm 2HL was a novel QTL specific for low N. Notably, Clusters C4 and C7 contained QTL for traits at both the seedling and maturity stages. In addition, four stable QTLs in Cluster C4 were identified. Furthermore, a gene (*HORVU2Hr1G080990.1*) related to grain protein in the interval of *Qtgw.sau-2H* was predicted. Correlation analysis and QTL mapping showed that different N treatments significantly affected agronomic and physiological traits at the seedling and maturity stages. These results provide valuable information for understanding N tolerance as well as breeding and utilizing the loci of interest in barley.

## 1. Introduction

Nitrogen (N) is one of the indispensable nutrient elements for plant growth and development [1]. The increase in crop yield depends mainly on the application of N fertilizer [2]. Over the past 40 years, the doubling of global grain production has been closely related to the surge in N application rates [3]. Excessive application of N fertilizer will decrease N use efficiency (NUE) and cause a series of environmental problems [4], such as global acidification, the destruction of stratospheric ozone and the eutrophication of water bodies [5]. As global soil waterlogging is expected to increase under the future climate, this will further lead to more N losses [6]. With the decrease in global planting area and the increase in food demand, cultivating crops with a high NUE plays a very important role in reducing the amount of N fertilizer applied and increasing grain yield (GY) [7].

Low N (LN) stress is a compound trait with a complex genetic basis. Many agronomic and physiological traits are involved in the response to LN stress in crops [8,9]. N deficiency will result in numerous adverse effects on the growth and development of crops. For example, it can phenotypically decrease plant height (PH), dry matter biomass and leaf area [10] and severely affect the root system’s architecture and yield-related traits [11]. It can also physiologically reduce the N content and a series of enzyme activities in the leaves [12]. However, some studies have shown that there are great differences in LN tolerance among different crop genotypes [13,14], which may provide a theoretical basis for breeding LN-tolerant cultivars.

QTL for agronomic and physiological traits have been mapped in several crops under N stress, including wheat [15,16,17,18,19], barley [20,21,22,23,24,25,26], maize [27,28,29] and rice [30,31]. For example, Saal et al. [20] detected 82 QTL for yield-related traits in two N treatments and six environments. Hoffmann et al. [22] located 58 QTL and identified beneficial wild barley alleles that improved the performance of several traits in response to different N treatments. Schnaithmann and Pillen [26] detected 65 QTL for 15 traits under different N treatments on seven chromosomes. Kindu et al. [25] detected 41 QTL on seven chromosomes for 18 traits in different treatments. Although some QTL for agronomic and physiological traits were identified, few of them were stably expressed, meaning that they were hard to utilize in breeding. More QTL for N tolerance under realistic barren soil conditions should be detected for marker-assisted selection (MAS) breeding.

Compared with other crops, barley is one of the earliest domesticated plants. It can grow under a wider range of conditions, especially under various stresses [32]. With the development of modern breeding and intensive farming, the genetic diversity of cultivated barley varieties has been reduced and many excellent alleles have been lost [33]. As a result, many cultivated barley varieties are sensitive to a variety of abiotic and biotic stress environments. Wild barley can maintain normal growth and productivity in soils with poor fertility. It has a rich genetic diversity and can provide excellent genes for breeding barley, thereby breaking the bottleneck of genetic improvement [34]. For example, Schmalenbach et al. [35] reported that 1000-grain weight (TGW) increased when wild barley alleles (*Hsp*) were introgressed into spring barley. Wild barley is generally better adapted to poor soil fertility. Therefore, we can assume that wild barley may contain some unique N tolerance genes. In this study, 39 agronomic and physiological traits were measured in a population of 121 recombinant inbred lines derived from the variety Baudin and the wild barley accession CN4027 in a hydroponic culture trial with low N (LN) and normal N (NN) treatments in a single timeframe, and in field trials with N deficiency (LN) and N sufficiency (NN) over two growing seasons, with the aim of uncovering favorable alleles for N stress tolerance in wild barley.

## 2. Results

### 2.1. Phenotypic Performance

For T1, 27 traits were investigated in total. Of these, 21 were root-related traits, including total root length (TRL, cm/plant), total root surface area (TRS, cm^2^/plant), total root volume (TRV, cm^3^/plant), average root diameter (ARD, cm/plant), total root tip number (TRTN), adventitious root length (ARL, cm/plant), adventitious root surface area (ARSA, cm^2^/plant), adventitious root volume (ARV, cm^3^/plant), adventitious root tip number (ARTN), lateral root length (LRL, cm/plant), lateral root surface area (LRSA, cm^2^/plant), lateral root volume (LRV, cm^3^/plant) and lateral root tip number (LRTN). The adventitious root length percentage (ARLP, ARL/TRL × 100%), adventitious root surface area percentage (ARSAP, ARSA/TRS × 100%), adventitious root volume percentage (ARVP, ARV/TRV × 100%), adventitious root tip number percentage (ARTNP, ARTN/TRTN × 100%), lateral root length percentage (LRLP, LRL/TRL × 100%), lateral root surface area percentage (LRSAP, LRSA/TRS × 100%), lateral root volume percentage (LRVP, LRV/TRV × 100%) and lateral root tip number percentage (LRTNP, LRTN/TRTN × 100%) were measured using the methods described by Ranoarisoa et al. [36].

Six growth-related traits, including shoot dry matter weight (SDW, g/plant) and root dry matter weight (RDW, g/plant), were measured using a 1/10,000 electronic balance. The dry root–shoot ratio (RSA, RDW/SDW) and total dry matter weight (TDW, SDW + RDW) were calculated. PH (cm/plant) was measured with a ruler. Leaf number (Ln) was recorded by counting the number of leaves longer than 2 cm 30 days after planting. The average values from three plants under LN and NN for each line were used for the following analyses.

For T1, the values for all phenotype traits except Ln were significantly influenced by different N treatments (Table 1). The SDW, RDW, TDW, PH, TRV, ARL and ARSA of CN4027 were significantly higher (*p* < 0.01) than those of Baudin at LN, whereas ARTN and ARTNP were significantly lower (*p* < 0.01). The TRL and ARTN of Baudin were significantly higher than CN4027 under NN (*p* < 0.01). There were no significant differences in RSA, Ln, TRS, ARD, LRL, LRSA, LRV, ARV, LRTN, LRLP, ARLP, LRSAP, ARSAP, LRVP, ARVP and LRTNP under the two N treatments. The coefficient of variation for the growth-related traits and root-related traits between the two N treatments ranged from 0.45 to 51.83% (Table 1). Among the RILs, transgressive segregation was observed for all traits (Table 1), indicating that they are typical quantitative traits.

For T2 and T3, 12 traits were evaluated, including five agronomic traits, namely PH, spike length (SL), awn length (AL, cm), growth period (GP) and lodging resistance (LDR), according to the Agricultural Industry Standards of the People’s Republic of China (NY/T 1301-2007). Seven yield-related traits were investigated, including grain number per spike (GN), spike number per plant (SN), GY, straw dry weight per plant (StDW), TGW (GY/GN*1000), aboveground dry weight per plant (ADW, GY + StDW) and harvest index (HI, GY/ADW). The average values of the three replications under the two different N treatments for each line were used for the following analyses. For T2 and T3, the phenotypic values for the Baudin, CN4027 and their RILs showed significant differences in most of the 12 investigated traits (Table 2). CN4027 had a significantly higher PH, AL, LDR, StDW, and ADW than Baudin under the two different N treatments, whereas SL, GP, GN, TGW, GY and HI were significantly lower (*p* < 0.01). The SN of CN4027 was significantly higher than that of Baudin under LN (*p* < 0.01). The coefficient of variation ranged from 1.77 to 56.85% for all traits. Among the RILs, transgressive segregation was also observed for all traits (Table 2).

### 2.2. Correlation Analysis between Investigated Traits

For T1, the correlations between growth- and root-related phenotypic traits under the two N treatments are presented in Table 3. Spearman’s correlations for growth- and root-related traits ranged from −0.17 to 0.98. Growth-related traits were significantly and positively correlated with all root-related traits under the NN treatment except for ARD, TRV and ARSA (*p* < 0.05). Under the LN treatment, significant and positive correlations were detected between root-related traits and both SDW and Ln (*p* < 0.01). TDW and PH were partly correlated with root-related traits (*p* < 0.05). However, RDW and RSR were not significantly correlated with root-related traits.

For T2 and T3, correlations between phenotypic agronomic traits and yield-related traits were listed in Table 4. Spearman’s correlation coefficient ranged from −0.81 to 0.92 (Table 4). Significant correlations were detected among each of the agronomic traits and most of the yield-related traits under both N conditions (*p* < 0.05). Under the LN treatment, agronomic traits, including SL, AL and LDR, were significantly and positively correlated with all the yield-related traits except StDW (Table 4). Under the NN treatment, SN, GN and TGW had significant positive correlations with SL, AL, LDR and GP (*p* < 0.05). Significant and positive correlations were detected for GY with PH, SL, AL and LDR and for StDW with PH, AL, LDR and GP (*p* < 0.05). ADW was significantly and positively correlated with all agronomic traits (*p* < 0.05). Consistently, significant and positive correlations were detected between HI and SL and between AL and GP (*p* < 0.01). Significant and negative correlations were detected between HI and both PH and LDR (*p* < 0.01) under the two N treatments at T2 and T3.

### 2.3. QTL for Seedling Traits

**Figure 1:**
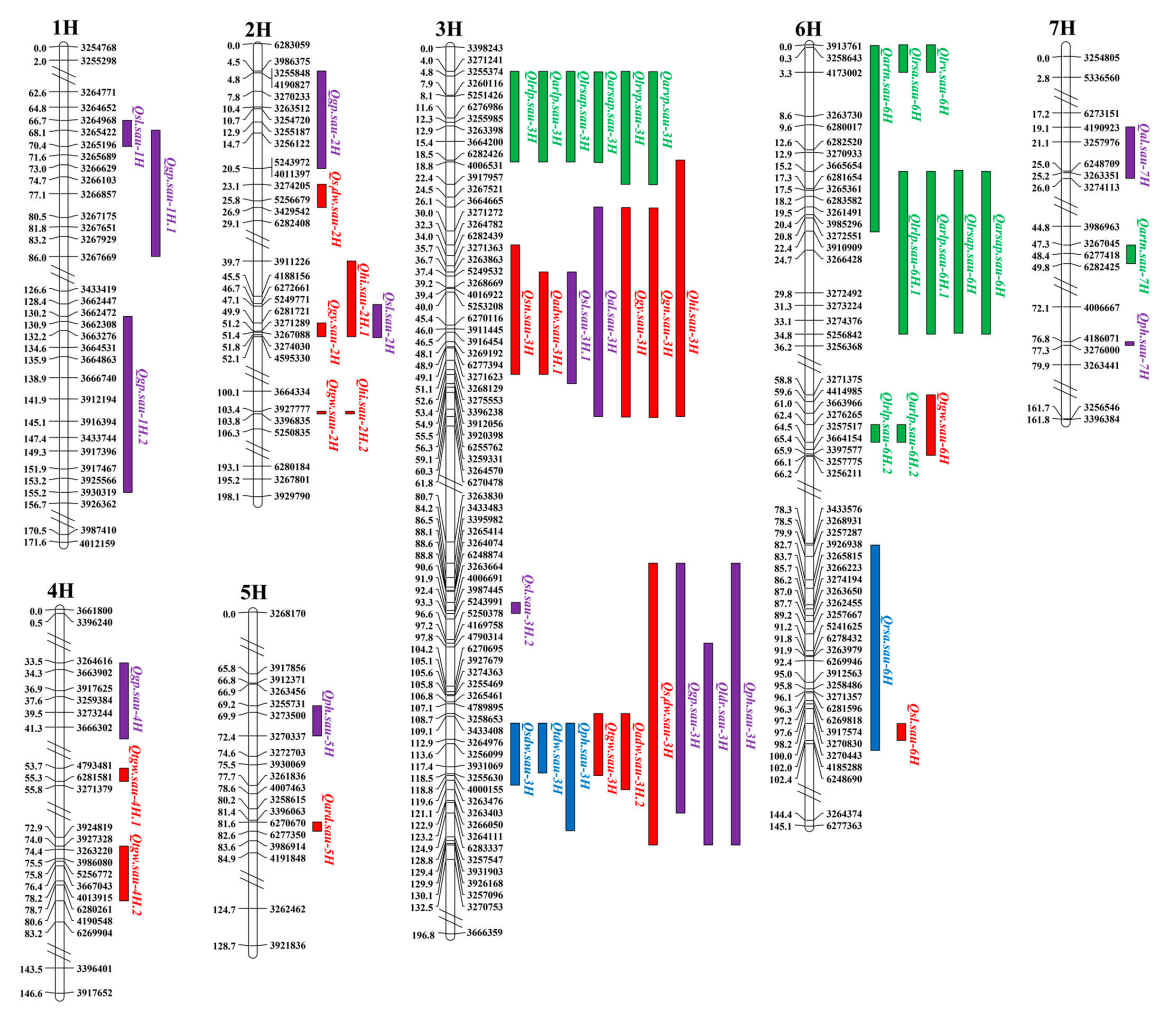
Quantitative trait loci (QTL) for agronomic and physiological traits identified at the seedling and maturity stages in the RIL population. Blue represents QTL conferring growth-related traits; green represents QTL conferring root-related traits; red represents QTL conferring yield-related traits; purple represents QTL conferring agronomic traits. 1H, chromosome 1H; 2H, chromosome 2H; 3H, chromosome 3H; 4H, chromosome 4H; 5H, chromosome 5H; 6H, chromosome 6H; 7H, chromosome 7H.

Twenty-one QTL for 27 traits, individually explaining 14.20–34.60% of the phenotypic variation rate (PVE) with a LOD value of 2.72–7.37, were detected. These were located on four chromosomes: 3H (9QTL), 5H (1), 6H (10) and 7H (1) (Figure 1 and Table 5). Positive alleles of 12 QTL were from CN4027, as well as 9 from Baudin. Interestingly, only *Qph.sau-3H* for PH, explaining 14.5–34.6% of the PVE, was detected under both LN and NN conditions, and its positive allele was derived from CN4027. Other QTL were detected under either the LN or NN treatment.

### 2.4. QTL for Maturity Traits

Thirty-two QTL for 12 maturity traits were detected and mapped on seven chromosomes (Figure 1 and Table 5): 1H (three QTL), 2H (seven), 3H (14), 4H (three), 5H (one), 6H (two) and 7H (two). They explained 12.10–62.20% of the PVE, with the LOD values ranging between 3.05 and 24.45. 

In total, 16 QTL for yield-related traits were detected, and the PVE explained by them ranged from 11.70% to 57.30% (Figure 1 and Table 5). The positive alleles of nine traits were contributed by Baudin, and those of the remaining seven traits were from CN4027. Of these, *Qgn.sau-3H* was detected on chromosome 3H, which explained 22.20% to 57.30% of the PVE under both N treatments in both T2 and T3. *Qtgw.sau-2H* was detected under LN in both T2 and T3, suggesting that it belongs to an LN-specific QTL. It explained up to 19.40% of the PVE, with a LOD value of 5.29. The positive allele of this trait was from CN4027*. Qgy.sau-3H*, which is located on 3H, was identified under both the LN and NN treatments in T2 and T3, explaining 19.30–37.50% of the PVE. *Qhi.sau-3H* was detected under all four N treatments, with a PVE of 35.90–50.50%.

For agronomic traits, 16 QTL were detected. The PVE explained by them was between 11.50% and 62.20% (Figure 1 and Table 5). The positive alleles of six traits were from Baudin, and 10 were from CN4027. *Qph.sau-3H* was detected under both N treatments in T2 and T3 and had the largest PVE (62.20%), with a LOD value of 24.45. Its positive allele was from CN4027. *Qal.sau-3H* was mapped to chromosome 3H under both N treatments in both trials conducted, explaining 12.3% to 30.10% of the PVE. *Qldr.sau-3H* was detected under four treatments and explained up to 35.80% of the PVE, with a LOD value of 11.27. *Qgp.sau-3H* was detected under the two different N treatments in T3 but only under the NN treatment in T2. with a PVE of 24.70% and a LOD value of 7.21. Its positive allele was derived from Baudin.

### 2.5. QTL Clusters

The QTL for different traits cluster together in a given interval, which is usually pleiotropic and important [37]. In this study, seven QTL clusters (C1–C7) were mapped to three chromosomes (2H, 3H and 6H). More than three QTL for each cluster were co-located (Table S3). These clusters could be classified into three types: those detected only at the seedling stage (Type I, including C2, C5 and C6) or the maturity stage (Type II, including C1 and C3), and those detected simultaneously at the seedling and maturity stages (Type III, including C4 and C7). Among them, Type I contained QTL only for root-related traits at the seedling stage. Notably, four LN-specific QTL for LPLP, LRSAP, ARSAP and ARLP were located in the region of 15.21–34.83 cM on chromosome 6H in C6. Type II contained QTL for both agronomic traits and yield-related traits at maturity. Four stable QTL (*Qgn.sau-3H*, *Qgy.sau-3H*, *Qhi.sau-3H* and *Qal.sau-3H*) in C3 were detected under four treatments and had higher PVE values (more than 12.30%). Type III contained QTL for traits at both seedling and maturity. Three of the 10 QTL in C4 were stable, namely *Qph.sau-3H*, *Qldr.sau-3H* and *Qgp.sau-3H*. They could be detected under all treatments and individually explained 12.30–62.20% of the PVE. In C7, QTL for LRLP and ARLP at the seedling stage and for TGW at maturity were co-located in the region of 58.84–66.07 cM on chromosome 6H.

## 3. Discussion

It has been shown that a limited N supply constrains crop production globally by influencing the crops’ growth and development [20,38]. For example, N deficiency can decrease barley yield, shorten the growth period and also decrease total biomass, the N content of organs and a series of enzyme activities [39,40,41]. Due to the complexity of physiological phenotypic characteristics, there is no standard evaluation index for a crop’s tolerance to LN stress. In this study, 39 traits under two N treatments at the seedling and maturity stages were measured to further evaluate the indexes of N tolerance in barley. Correlation analysis showed that different N treatments strongly affected the correlations among different types of traits at the seedling and maturity stages. In addition, the seven QTL clusters identified in this study provided strong evidence that the genetic mechanisms of growth-related traits, root-related traits, agronomic traits and yield-related traits in barley were closely related under different N treatments. In conclusion, the correlation analysis and QTL mapping showed that there was a close relationship between the four N tolerance evaluation indexes in barley under the different N treatments.

Under LN stress, plants may adopt different strategies to cope with the change in N [42]. In comparison with cultivated barley, wild barley has wider genetic diversity and is better adapted to N deficiency [43]. Identification of the resources of wild barley with higher tolerance to LN will provide excellent genetic material for improving LN tolerance in barley and other cereal crops [42]. QTL analysis suggested that the major *Qtgw.sau-2H* was stably expressed under only the LN treatment and the positive allele was from wild barley (CN4027). We speculate that it is an LN-sensitive QTL and has the potential for breeding to develop barley varieties with LN tolerance.

Roots, as the most important organ for N absorption, can adapt to changes in the N supply by changing their morphology over time [44]. In this study, Baudin had a larger LRLP and LRSAP, while CN4027 had a larger ARLP and ARSAP under LN. Thus, the characterization and aggregation of LN-specific QTL in different varieties may be helpful for breeding high-NUE varieties [45]. Remarkably, there was a co-localized region for root-related traits under LN. *Qlrlp.sau-6H.1*, *Qarlp.sau-6H.1*, *Qlrsap.sau-6H* and *Qarsap.sau-6H* were co-located between *bpb-3665654* and *bpb-5256842* on chromosome arm 6HS, indicating that the favorable alleles for this region may increase N absorption capacity by changing root systems and root phenotypes. Eight stably expressed QTL for traits identified at maturity under more than three treatments were obtained in this study. To confirm whether the newly identified QTL were novel loci, we compared their physical intervals with previously reported QTL through anchoring their flanking markers on barley using BLASTn analyses against the Morex genome database. *Qgy.sau-3H*, *Qhi.sau-3H*, *Qgn.sau-3H* and *Qal.sau-3H* were mapped in the interval between 16.54 and 435.82 Mb on chromosome 3H. This interval overlapped with QTL for GY on 3H (345.20 Mbp) [46], with QTL for AL [47] and a QTL hotspot for yield-related traits [48]. In addition, *Qph.sau-3H*, *Qldr.sau-3H* and *Qgp.sau-3H* were identified in the interval between 473.84 and 528.17 Mb on chromosome arm 3HL, which overlapped with the *sdw1*/*denso* genes for PH [49,50]. These results suggested that they may not be novel loci.

An LN-specific *Qtgw.sau-2H* for TGW was located in a 0.46 cM interval and physically mapped between 524.23 and 524.63 Mbp on chromosome arm 2HL. Few QTL-controlling TGW have been reported on chromosome 2H. For example, Wang et al. [48] identified some QTL for grain length, grain width, grain length–width ratio, TGW, grain area, grain perimeter, grain diameter, grain roundness and factor form density between 647.83 and 653.98 Mbp on chromosome arm 2HL. Wang et al. [51] detected *qTgw2-1* and *qTgw2-2* for TGW on 2HL, which were close to the morphological marker *Vrs1* (690.31 Mbp). These previously reported QTL were unlikely to be allelic to the *Qtgw.sau-2H* locus identified in this study, suggesting that *Qtgw.sau-2H* might be a novel locus.

In the present study, *Qtgw.sau-2H* was located between the markers *bpb3927777* and *bpb3396835*. Only one candidate gene was predicted in this region (Table S4). The co-located intervals for *Qgy.sau-3H*, *Qhi.sau-3H*, *Qgn.sau-3H* and *Qal.sau-3H* were located between the markers *bpb6282426* and *bpb3264570*; there were eight candidate genes in this region. The intervals for *Qph.sau-3H*, *Qldr.sau-3H* and *Qgp.sau-3H* were co-located between markers *bpb3433483* and *bpb3257096*; there were 12 candidate genes in this interval (Table S4). Among these were candidate genes in each interval for the QTL. For example, *HORVU2Hr1G080990.1* encodes a seed storage 2S albumin superfamily protein. A large amount of Seed storage 2S albumin superfamily protein is synthesized at the late stage of seed development [52,53]. The protein content and the degradation rate of stored protein are closely related to seed vigor [54]. *HORVU3Hr1G091720.2* encodes a NAC domain-containing protein, and it plays a regulatory role in the growth and development of millet [55]. *HORVU3Hr1G091460.10* encodes an SET domain-containing protein that has been reported to play a role in nutrient regulation under abiotic stresses [56]. *HORVU3Hr1G061710.1* and *HORVU3Hr1G089840.1* encode an L-asparaginase that has been reported to affect NUE and GY in Arabidopsis [57,58]. *HORVU3Hr1G063180.1* encodes glutamate dehydrogenase 2, which has been reported to improve NUE traits [23]. *HORVU3Hr1G066050.1* and *HORVU3Hr1G093310.2* encode the transcription factor basic helix-loop-helix 62 (bHLH62), which has been reported to participate in the transport of NH4^+^ [59].

N stress in the soil is becoming a huge problem for crop production around the world. It is urgent to develop crop cultivars with strong LN tolerance for coping with the problem. A wild barley allele introgression of S42IL-119 was associated with a 20% increase in TKW under LN conditions [26]. Quan et al. [60] showed that soluble acid invertase, MYB and NAC transcription factors, ethylene biosynthesis and oxygen species play an important role in the LN tolerance of barley. Meanwhile, agronomic and physiological traits are often used as indicators of plants’ tolerance to LN stress [61,62,63]. In this study, eight stable QTL were identified that were related to agronomic and physiological traits, which provide clues for developing barley with LN tolerance.

QTL clusters containing stable QTL are particularly important [64]. In this study, two QTL clusters (C3 and C4) contained stable QTL. C3 on chromosome 3H contained four stable QTL (*Qgn.sau-3H*, *Qgy.sau-3H*, *Qhi.sau-3H* and *Qal.sau-3H*). The most important cluster, C4 on chromosome 3H, included 3 and 7 QTL for seedling and maturity traits, respectively. Interestingly, *Qph.sau-3H* and *Qldr.sau-3H* were co-localized between the markers *bpb3433483* and *bpb3257096*, which showed the existence of a close genetic relationship between PH and LDR [65]. The markers in these two QTL clusters should be helpful for marker-assisted selection in barley breeding programs under LN stress. The QTL clusters could provide important information for selecting genotypes with better early vigor and N uptake efficiency.

## 4. Materials and Methods

### 4.1. Plant Materials

An F_8_ RIL population, including 121 lines, was constructed using the single-seed descent method from the cross between the barley cultivar Baudin and the wild accession CN4027 [66]. Baudin is a high-yielding variety that can adapt to longer seasons and regions with moderate rainfall; CN4027 is a wild barley genotype.

### 4.2. Hydroponic Trial

A hydroponic trial (T1) was adopted at the seedling stage with two N concentrations provided using Ca(NO_3_)_2_·4H_2_O at 2 mmol·L^−1^ (NN) and 0.5 mmol·L^−1^ (LN) [22]. Twenty seeds with a similar size from each line in the BC population were selected and disinfected with 10% H_2_O_2_ for 5 min, then cleaned with distilled water and germinated at 25 °C and 60% humidity. Three seedlings from each line with consistent growth were transferred to a plastic tank (60 × 40 × 15 cm^3^) containing Hoagland nutrient solution (Table S1) at the two-leaf stage. The nutrient solution was changed every 5 days, and ventilation was conducted every 2 h. The pH of the nutrient solution was adjusted to 5.5–6.0 with 0.1 mol·L^−1^ HCl or NaOH. Each treatment was repeated three times.

T1 was conducted in the rainproof greenhouse facilities of the Chengdu Campus of Sichuan Agricultural University from September to November in 2019. After transplantation, the samples were collected after being treated with different N concentrations for 30 days. The samples were washed with tap water, moistened with distilled water and then dried with absorbent paper. Epson Expression 10,000 XL was used for collecting images of the roots. Win RHIZO-Pro V2007d (Regent Instruments Inc., Quebec, QC, Canada) was used to analyze the root-related traits. The Ln was calculated for leaves measuring more than 0.2 cm at harvest. PH was measured from the base of the plant to the top. The shoot and root tissues were packed in bags and placed in an oven for deactivation at 105 °C for 30 min and dried at 75 °C until they had a constant weight. The RDW (g/plant) and SDW (g/plant) were measured using a 1/10,000 electronic balance. The dry root–shoot ratio (DRS, RDW/SDW) and TDW (RDW + SDW) were calculated.

### 4.3. Field Trials

The field trials were conducted using a split-plot design with the N treatments (normal N (NN) and no N (LN)) as the main plot and the genotypes as the subplots. The two main plots were separated by a trench 60 cm deep. Pure N at 150 kg·hm^−2^, with the N source being urea (N, 46%), was adopted as the normal N treatment. In both trials, pure phosphorus (P_2_O_5_ 75 kg·hm^−2^), superphosphate (P_2_O_5_, 12%), pure potassium (K_2_O 75 kg hm^−2^) and potassium chloride (K_2_O, 60%) were applied. The trials were conducted at Shifang and Deyang in Sichuan Province during the growing seasons of 2020–2021 (T2) and 2021–2022 (T3). These two experiment fields belong to the mid-latitude of the subtropical humid climate zone. The type of soil in the experimental field is paddy soil, and the basic physical and chemical properties of soil before planting are shown in Table S2. Each line was planted as a single seed in two rows 0.8 m in length, with 6 cm between plants within a row and 20 cm between the rows. Each row was treated as a replicate, and each treatment was repeated three times. The fields were managed by conventional practices for barley production. We collected the aerial parts of five plants with similar growth conditions for each of the three repetitions at the mature stage, then numbered and bagged them. We then rinsed the samples with distilled water and finally dried them with absorbent paper. The straw dry weight per plant and spike weight was measured using an electronic balance after the straw and spike tissues had been deactivated at 105 °C for half an hour and then dried to a constant weight at 75 °C.

### 4.4. QTL Mapping and Statistical Analysis

The genetic linkage map reported in an earlier study [57] was used for mapping the QTL. Spearman’s correlation coefficients among traits at seedling and maturity stages were calculated using SPSS 22.0 (SPSS Inc., Chicago, IL, USA). Excel (2016) was used for drawing the charts. Interval mapping in MapQTL5.0 was used for mapping the QTL with LOD > 2.5 at the seedling stage and LOD > 3.0 at maturity according to the standards of previous studies [19,58], and MapChart2.0 software was used for map construction. QTL were named following the recommendations of the International Rules of Genetic Nomenclature (http://wheat.pw.usda.gov/ggpages/wgc/98/Intro.htm (accessed on 15 April 2022)).

## 5. Conclusions

In our study, we identified eight stable QTL for agronomic and physiological traits, including GN, TGW, GY, HI, PH, AL, LDR and GP under different N treatments. Thirty-five QTL formed seven QTL clusters on chromosomes 2H, 3H and 6H. Additionally, we evaluated the relationships among growth-related traits and root-related traits, agronomic traits and yield-related traits. A novel stable LN-specific QTL for TGW was identified on chromosome 2H under the LN treatments. A predicted gene (*HORVU2Hr1G080990.1*) in the *Qtgw.sau-2H* interval may be valuable for subsequent fine mapping of candidate genes. These results provide valuable information for understanding N tolerance as well as breeding and utilizing the loci of interest in barley.

## Figures and Tables

**Table 1:** Variations in the traits evaluated in the RIL population and their parents at seedling stage.

Types	Trait	Treatment	T1				
			Parents		RIL Population		
			Baudin	CN4027	Range	Mean ± SD	CV(%)
Growth-related traits	SDW	LN	0.03	0.06 **	0.02–0.12	0.07 ± 0.02	33.62
		NN	0.06	0.08	0.03–0.15	0.10 ± 0.03	28.20
	RDW	LN	0.02	0.04 **	0.01–0.07	0.03 ± 0.01	40.66
		NN	0.02	0.02	0.01–0.04	0.02 ± 0.01	33.10
	TDW	LN	0.04	0.10 **	0.02–0.17	0.10 ± 0.03	33.62
		NN	0.08	0.10	0.03–0.19	0.12 ± 0.04	28.50
	RSA	LN	51.72	59.02	20.00–90.72	46.06 ± 11.52	25.01
		NN	30.16	24.05	14.13–38.09	24.75 ± 4.43	17.88
	PH	LN	22.10	34.30 **	12.10–46.80	33.60 ± 6.60	19.74
		NN	36.60	36.50	25.40–52.20	38.70 ± 5.30	13.80
	Ln	LN	4.00	4.00	3.00–6.00	4.00 ± 1.00	25.00
		NN	5.00	4.00	4.00–8.00	5.00 ± 1.00	20.00
Root-related traits	TRL	LN	409.50	681.10	57.50–1703.60	837.50 ± 368.20	43.97
		NN	231.10	434.80 **	125.00–911.50	476.20 ± 181.40	38.09
	TRS	LN	32.90	58.20	5.70–134.90	63.80 ± 28.10	44.09
		NN	20.80	37.30	10.70–71.20	42.30 ± 15.00	35.45
	ARD	LN	0.31	0.31	0.23–0.41	0.28 ± 0.02	8.42
		NN	0.35	0.32	0.28–0.42	0.34 ± 0.03	7.91
	TRV	LN	0.34	0.61 **	0.10–1.41	0.61 ± 0.28	45.38
		NN	0.26	0.42	0.11–0.87	0.49 ± 0.17	34.54
	TRTN	LN	871.00	932.00	122.00–3251.00	1339.00 ± 647.00	48.28
		NN	270.00	489.00 **	163.00–1442.00	670.00 ± 275.00	41.09
	LRL	LN	307.90	438.00	44.50–1290.00	625.70 ± 279.60	44.68
		NN	166.00	306.60	85.50–660.10	315.50 ± 132.20	41.91
	ARL	LN	101.60	243.10 **	13.10–591.40	211.70 ± 105.30	49.73
		NN	65.10	128.20	25.00–321.50	160.70 ± 62.40	38.81
	LRSA	LN	17.10	20.90	2.50–60.70	30.70 ± 13.10	42.68
		NN	8.60	15.70	5.00–32.40	16.40 ± 6.50	39.56
	ARSA	LN	15.80	37.30 **	3.20–90.50	33.10 ± 17.20	51.83
		NN	12.20	21.60	4.00–50.10	26.00 ± 9.70	37.21
	LRV	LN	0.09	0.10	0.01–0.27	0.14 ± 0.06	41.02
		NN	0.04	0.07	0.03–0.15	0.08 ± 0.03	38.07
	ARV	LN	0.25	0.51	0.07–1.22	0.47 ± 0.23	48.99
		NN	0.22	0.34	0.07–0.73	0.41 ± 0.15	36.06
	LRTN	LN	859.00	928.00	118.00–3232.00	1326.00 ± 643.00	48.49
		NN	263.00	474.00	160.00–1420.00	658.00 ± 273.00	41.52
	ARTN	LN	12.00 **	4.00	1.00–30.00	13.00 ± 6.00	46.57
		NN	7.00	15.00 **	3.00–26.00	12.00 ± 5.00	41.17
	LRLP	LN	75.20	64.30	59.20–88.00	75.30 ± 6.30	8.33
		NN	71.80	70.50	51.70–80.40	65.80 ± 6.70	10.19
	ARLP	LN	24.80	35.70	13.40–41.60	24.80 ± 6.30	25.29
		NN	28.20	29.50	19.60–48.30	34.20 ± 6.70	19.60
	LRSAP	LN	52.00	35.90	17.70–70.40	49.80 ± 9.10	18.35
		NN	41.20	42.20	24.00–62.70	38.80 ± 7.00	18.14
	ARSAP	LN	48	64.1	29.60–82.30	50.20 ± 9.10	18.18
		NN	58.8	57.8	37.30–76.00	61.20 ± 7.00	11.52
	LRVP	LN	26.7	15.9	11.10–42.20	24.90 ± 6.70	26.72
		NN	15.4	17.7	10.20–31.10	16.80 ± 4.70	27.82
	ARVP	LN	73.3	84.1	57.80–88.90	75.10 ± 6.70	8.85
		NN	84.6	82.3	62.70–91.80	83.10 ± 5.00	6.07
	LRTNP	LN	98.6	99.6	97.40–99.50	98.90 ± 0.40	0.45
		NN	97.4	96.9	94.90–99.50	97.90 ± 0.90	0.94
	ARTNP	LN	1.4 **	0.4	0.30–2.60	1.10 ± 0.50	46.80
		NN	2	3.1	0.50–5.10	2.10 ± 0.90	44.86

LN, low nitrogen; NN, normal nitrogen; CV, coefficient of variation; SDW, shoot dry matter weight; RDW, root dry matter weight; TDW, total dry matter weight; RSA, root–shoot ratio; PH, plant height; Ln, leaf number; TRL, total root length; TRV, total root volume; ARD, average root diameter; TRTN, total root tip number; ARL, adventitious root length; ARSA, adventitious root surface area; ARV, adventitious root volume; ARTN, adventitious root tip number; ARLP, adventitious root length percentage; ARSAP, adventitious root surface area percentage; ARVP, adventitious root volume percentage; ARTNP, adventitious root tip number percentage; T, Trial. ** Mean significant difference at *p* < 0.01.

**Table 2:** Variations of traits evaluated in the RIL population and their parents at maturity.

Types	Trait	Treatment	T2					T3				
			Parents		RIL Population			Parents		RIL Population		
			Baudin	CN4027	Range	Mean ± SD	CV(%)	Baudin	CN4027	Range	Mean ± SD	CV(%)
Agronomic traits	PH	LN	61.26	104.77 **	52.08–126.83	86.54 ± 19.54	22.58	55.06	103.24 **	56.00–130.30	85.55 ± 18.10	21.16
		NN	72.95	112.3 **	64.24–132.70	97.74 ± 18.73	19.16	70.15	119.60 **	66.63–133.05	95.76 ± 18.27	19.08
	SL	LN	9.95 **	8.55	4.33–12.27	8.88 ± 1.51	17.00	9.10 **	7.52	4.50–13.03	8.57 ± 1.03	12.02
		NN	10.58 **	9.75	7.14–12.95	10.06 ± 1.12	11.13	9.63 **	8.55	5.42–10.86	8.14 ± 1.06	13.02
	AL	LN	7.98	7.68	5.16–13.54	8.82 ± 1.78	20.18	9.28	11.32 **	1.14–13.18	9.60 ± 1.54	16.04
		NN	9.52	12.4 **	5.80–16.32	10.06 ± 1.73	17.20	8.98	10.68 **	6.55–14.60	10.02 ± 1.55	15.47
	LDR	LN	1.67	4.33 **	1.00–5.00	3.66 ± 0.74	20.22	1.33	5.00 **	1.00–5.00	2.73 ± 0.69	25.27
		NN	1.67	4.67 **	1.00–5.00	3.08 ± 0.75	24.35	1.67	4.67 **	1.00–5.00	2.85 ± 0.85	29.82
	GP	LN	174.33 **	165.33	159.00–178.33	168.69 ± 2.98	1.77	168.33 **	162.67	159.33–179.00	165.54 ± 5.28	3.19
		NN	176.67 **	168.67	162.67–181.33	170.96 ± 3.43	2.01	174.33 **	167.33	162.00–180.67	168.18 ± 4.90	2.91
Yield-related traits	SN	LN	8.67	16.44 **	6.56–28.22	14.76 ± 4.84	32.78	7.56	18.00 **	3.56–27.56	12.12 ± 4.61	38.02
		NN	19.89	20.22	5.00–46.33	18.44 ± 7.62	41.31	20.11	21.33	4.00–33.56	15.44 ± 5.95	38.51
	GN	LN	22.44 **	7.44	3.67–25.11	14.72 ± 5.30	36.01	21.89 **	6.78	1.89–36.33	13.67 ± 5.51	40.31
		NN	21.00 **	11.56	4.78–55.89	16.03 ± 6.64	41.43	17.78 **	9.22	2.89–41.67	14.54 ± 6.58	45.27
	TGW	LN	47.52 **	35.23	23.84–53.58	37.09 ± 5.71	15.39	45.78 **	26.70	25.08–51.30	36.95 ± 4.50	12.17
		NN	47.00 **	26.30	26.52–53.22	39.06 ± 5.61	14.35	33.45 **	29.08	19.34–48.62	34.39 ± 5.60	16.28
	GY	LN	8.41 **	4.14	1.35–26.34	7.94 ± 4.19	52.77	7.39 **	2.97	0.50–19.64	6.07 ± 3.45	56.85
		NN	20.19 **	6.53	1.43–33.41	11.71 ± 6.14	52.43	11.89 **	5.58	1.26–17.71	7.57 ± 4.17	55.14
	HI	NN	0.56 **	0.23	0.12–0.76	0.39 ± 0.13	32.52	0.57 **	0.16	0.09–0.70	0.41 ± 0.13	31.83
		NN	0.60 **	0.23	0.12–0.73	0.43 ± 0.12	27.99	0.52 **	0.15	0.10–0.61	0.38 ± 0.12	32.02
	StDW	LN	5.65	15.60 **	2.02–20.60	8.65 ± 4.17	48.26	5.65	15.60 **	2.02–20.60	8.65 ± 4.17	48.26
		NN	10.96	31.10 **	1.92–34.72	12.12 ± 5.96	49.19	10.96	31.10 **	1.92–34.72	12.12 ± 5.96	49.19
	ADW	LN	13.04	18.57 **	2.52–37.49	14.74 ± 6.46	43.86	13.04	18.57 **	2.52–37.49	14.74 ± 6.46	43.86
		NN	22.86	36.69 **	3.58–49.93	19.70 ± 8.56	43.47	22.86	36.69 **	3.58–49.93	19.70 ± 8.56	43.47

LN, no applied nitrogen; NN, normal nitrogen; CV, coefficient of variation; PH, plant height; SL, spike length; AL, awn length; LDR, lodging resistance; GP, growth period; SN, spike number per plant; GN, grain number per spike; TGW, 1000-grain weight; GY, grain yield; HI, harvest index; StDW, straw dry weight per plant; ADW, aboveground dry weight per plant; T, trial. ** Mean significant difference at *p* < 0.01.

**Table 3:** Correlations between growth- and root-related traits in the RIL population at the seedling stage.

Trial	Traits	Treatments	TRL	TRS	ARD	TRV	TRTN	LRL	LRSA	LRV	LRTN	ARL	ARSA	ARV	ARTN
T1	SDW	LN	0.30 **	0.63 **	0.62 **	0.65 **	0.67 **	0.46 **	0.40 **	0.370 **	0.67 **	0.73 **	0.67 **	0.61 **	0.57 **
		NN	0.97 **	0.98 **	0.92 **	0.98 **	0.93 **	0.94 **	0.95 **	0.95 **	0.93 **	0.96 **	0.97 **	0.98 **	0.92 **
	RDW	LN	−0.12	−0.07	−0.13	−0.13	−0.06	−0.10	−0.14	−0.08	−0.06	−0.10	−0.08	−0.13	−0.11
		NN	0.73 **	0.72 **	0.70 **	0.70 **	0.72 **	0.73 **	0.72 **	0.72 **	0.73 **	0.74 **	0.72 **	0.71 **	0.73 **
	RSR	LN	−0.11	−0.11	−0.16	−0.16	−0.12	−0.17	−0.13	−0.07	−0.12	−0.15	−0.14	−0.16	−0.15
		NN	0.59 **	0.58 **	0.61 **	0.60 **	0.60 **	0.60 **	0.60 **	0.61 **	0.60 **	0.63 **	0.60 **	0.60 **	0.62 **
	TDW	LN	0.03	0.24 *	0.18	0.19	0.27 *	0.13	0.07	0.11	0.26 *	0.26 *	0.25 *	0.18	0.18
		NN	0.78 **	0.79 **	0.75 **	0.77 **	0.77 **	0.77 **	0.77 **	0.77 **	0.77 **	0.79 **	0.78 **	0.77 **	0.77 **
	PH	LN	0.21	0.51 **	0.30 **	0.33 **	0.33 **	0.38 **	0.34 **	0.35 **	0.33 **	0.37 **	0.26 *	0.23 *	0.28 **
		NN	0.51 **	0.53 **	0.48 **	0.52 **	0.47 **	0.48 **	0.49 **	0.50 **	0.47 **	0.52 **	0.53 **	0.52 **	0.49 **
	Ln	LN	0.40 **	0.71 **	0.57 **	0.66 **	0.79 **	0.54 **	0.53 **	0.57 **	0.79 **	0.61 **	0.68 **	0.63 **	0.52 **
		NN	0.25 *	0.22 *	0.21	0.20	0.28 *	0.25 *	0.22 *	0.23 *	0.28 *	0.23 *	0.19	0.21 *	0.27 *

LN, low nitrogen; NN, normal nitrogen; SDW, shoot dry matter weight; RDW, root dry matter weight; RSR, root–shoot ratio; TDW, total dry matter weight; PH, plant height; Ln, leaf number; TRL, total root length; TRS, total root surface area; ARD, average root diameter; TRV, total root volume; TRTN, total root tip number; LPL, lateral root length; LRSA, lateral root surface area; LRV, lateral root volume; LRTN, lateral root tip number; ARL, adventitious root length; ARSA, adventitious root surface area; ARV, adventitious root volume; ARTN, adventitious root tip number; T, trial. * and ** mean significant differences at *p* < 0.05 and 0.01, respectively.

**Table 4:** Correlations among agronomic traits and yield-related traits in the RIL population at maturity.

Trials	Traits	Treatments	SN	GN	TGW	GY	StDW	ADW	HI
T2	PH	LN	0.54 **	−0.26 **	−0.23 *	0.38 **	0.20 *	0.22 *	−0.29 **
		NN	0.12	0.07	0.11	0.19 *	0.38 **	0.23 *	−0.57 **
	SL	LN	0.67 **	0.27 **	0.56 **	0.67 **	0.10	0.21 *	0.57 **
		NN	0.63 **	0.50 **	0.74 **	0.53 **	0.65 **	0.66 **	0.63 **
	AL	LN	0.92 **	−0.73 **	0.38 **	0.82 **	0.92 **	0.92 **	0.32 **
		NN	0.80 **	0.67 **	0.88 **	0.70 **	0.80 **	0.79 **	0.55 **
	LDR	LN	0.39 **	0.37 **	0.45 **	0.33 **	0.41 **	0.36 **	−0.26 **
		NN	0.62 **	0.47 **	0.49 **	0.57 **	0.66 **	0.66 **	−0.27 **
	GP	LN	0.82 **	−0.63 **	0.27 **	0.73 **	0.81 **	0.84 **	0.24 **
		NN	0.27 **	0.28 **	0.39 **	0.12	0.23 *	0.23 *	0.71 **
T3	PH	LN	0.46 **	−0.34 **	0.11	0.61 **	0.29 **	0.24 *	−0.40 **
		NN	0.08	−0.11	−0.18	0.17 *	0.26 **	0.34 **	−0.51 **
	SL	LN	0.67 **	0.39 **	0.39 **	0.59 **	0.17	0.24 *	0.32 **
		NN	0.20 *	0.40 **	0.65 **	0.34 **	0.06	0.19 *	0.61 **
	AL	LN	0.44 **	−0.81 **	0.46 **	0.34 **	0.20 *	0.26 **	0.25 **
		NN	0.29 **	0.55 **	0.72 **	0.21 *	0.24 *	0.32 **	0.55 **
	LDR	LN	0.67 **	0.33 **	0.35 **	0.63 **	0.50 **	0.44 **	−0.21 *
		NN	0.45 **	0.20 *	0.49 **	0.49 **	0.43 **	0.52 **	−0.37 **
	GP	LN	0.50 **	−0.73 **	0.21 *	0.60 **	0.48 **	0.36 **	0.29 **
		NN	0.24 **	0.18 *	0.31 **	−0.08	0.24 **	0.16 *	0.22 *

LN, no applied nitrogen; NN, normal applied nitrogen; PH, plant height; SL, spike length; AL, awn length; LDR; lodging resistance; GP, growth period; SN, spike number per plant; GN, grain number per spike; TGW, 1000-grain weight; GY, grain yield; StDW, straw dry weight per plant; ADW, aboveground dry weight per plant; HI, harvest index; T, trial. * and **, mean significant difference at *p* < 0.05 and 0.01.

**Table 5:** Quantitative trait loci (QTL) for agronomic and physiological traits identified at the seedling and maturity stages.

Types	Trait	QTL	Chr.	Treatment	Marker Interval	LOD	PVE (%)	Origin
Growth-related traits	RSA	*Qrsa.sau-6H*	6H	T1LN	*bpb3268931*–*bpb6273107*	3.39	16.10	Baudin
	SDW	*Qsdw.sau-3H*	3H	T1NN	*bpb3264976*–*bpb3264111*	4.42	22.50	CN4027
	TDW	*Qtdw.sau-3H*	3H	T1NN	*bpb3264976*–*bpb3263403*	4.35	22.20	CN4027
	PH	*Qph.sau-3H*	3H	T1LN	*bpb3264976*–*bpb3257096*	3.02	14.50	CN4027
				T1NN		7.37	34.60	CN4027
Root-related traits	ARD	*Qard.sau-5H*	5H	T1NN	*bpb6270670*–*bpb6277350*	2.80	14.90	Baudin
	LRSA	*Qlrsa.sau-6H*	6H	T1NN	*bpb3266659*–*bpb4173002*	2.74	14.60	CN4027
	LRV	*Qlrv.sau-6H*	6H	T1NN	*bpb3266659*–*bpb4173002*	2.72	14.50	CN4027
	ARTN	*Qartn.sau-7H*	7H	T1LN	*bpb3267045*–*bpb6282425*	2.94	14.20	Baudin
		*Qartn.sau-6H*	6H	T1NN	*bpb3266659*–*bpb3910909*	3.02	16.20	CN4027
	LRLP	*Qlrlp.sau-6H.1*	6H	T1LN	*bpb3665654*–*bpb5256842*	4.28	19.90	Baudin
		*Qlrlp.sau-6H.2*	6H	T1NN	*bpb3276265*–*bpb3257517*	3.74	20.10	Baudin
		*Qlrlp.sau-3H*	3H	T1NN	*bpb3271241*–*bpb4006531*	5.03	25.10	Baudin
	ARLP	*Qarlp.sau-6H.1*	6H	T1LN	*bpb3665654*–*bpb5256842*	4.28	19.90	CN4027
		*Qarlp.sau-6H.2*	6H	T1NN	*bpb3276265*–*bpb3257517*	3.74	20.10	CN4027
		*Qarlp.sau-3H*	3H	T1NN	*bpb3271241*–*bpb4006531*	5.03	25.10	CN4027
	LRSAP	*Qlrsap.sau-6H*	6H	T1LN	*bpb3665654*–*bpb5256842*	3.97	18.60	Baudin
		*Qlrsap.sau-3H*	3H	T1NN	*bpb3271241*–*bpb4006531*	5.23	26.00	Baudin
	ARSAP	*Qarsap.sau-6H*	6H	T1LN	*bpb3665654*–*bpb5256842*	3.97	18.60	CN4027
		*Qarsap.sau-3H*	3H	T1NN	*bpb3271241*–*bpb4006531*	5.23	26.00	CN4027
	LRVP	*Qlrvp.sau-3H*	3H	T1NN	*bpb3271241*–*bpb3917957*	3.89	20.10	Baudin
	ARVP	*Qarvp.sau-3H*	3H	T1NN	*bpb3271241*–*bpb3917957*	3.89	20.10	CN4027
Yield-related traits	SN	*Qsn.sau-3H*	3H	T2NN	*bpb3264782*–*bpb3396238*	4.91	17.80	Baudin
	GN	*Qgn.sau-3H*	3H	T2LN	*bpb3664665*–*bpb3264570*	18.91	57.30	Baudin
				T3LN		8.65	29.30	Baudin
				T2NN		6.20	22.20	Baudin
				T3NN		8.52	29.20	Baudin
	TGW	*Qtgw.sau-2H*	2H	T2LN	*bpb3927777*–*bpb3396835*	3.57	13.80	CN4027
				T3LN		5.29	19.40	CN4027
		*Qtgw.sau-3H*	3H	T3NN	*bpb3258653*–*bpb4000155*	3.78	14.10	CN4027
		*Qtgw.sau-4H.1*	4H	T2LN	*bpb4793481*–*bpb6281581*	3.37	12.90	Baudin
		*Qtgw.sau-4H.2*	4H	T2LN	*bpb3927328*–*bpb4190548*	3.55	13.60	Baudin
		*Qtgw.sau-6H*	6H	T2NN	*bpb3271375*–*bpb3257775*	5.51	19.60	Baudin
	GY	*Qgy.sau-2H*	2H	T2LN	*bpb6281721*–*bpb4595330*	4.25	15.60	CN4027
		*Qgy.sau-3H*	3H	T2LN	*bpb3664665*–*bpb3264570*	9.58	33.10	Baudin
				T3LN		5.41	19.30	Baudin
				T2NN		11.40	37.50	Baudin
				T3NN		7.62	26.50	Baudin
	StDW	*Qs_t_dw.sau-2H*	2H	T3LN	*bpb3274205*–*bpb3429542*	3.86	14.30	CN4027
		*Qs_t_dw.sau-3H*	3H	T3LN	*bpb3433483*–*bpb3257096*	3.49	13.10	CN4027
				T3NN		5.62	20.30	CN4027
	TDW	*Qadw.sau-3H.1*	3H	T2NN	*bpb3263863*–*bpb3396238*	5.70	21.00	Baudin
		*Qadw.sau-3H.2*	3H	T3NN	*bpb3258653*–*bpb3263403*	3.85	14.50	CN4027
	HI	*Qhi.sau-2H.1*	2H	T3-N	*bpb3911226*–*bpb4595330*	4.42	16.20	CN4027
				T3NN		3.05	11.70	CN4027
		*Qhi.sau-2H.2*	2H	T3LN	*bpb3927777*–*bpb3396835*	3.54	13.30	Baudin
		*Qhi.sau-3H*	3H	T2LN	*bpb6282426*–*bpb3264570*	17.39	50.50	Baudin
				T3LN		12.53	40.60	Baudin
				T2NN		12.15	38.30	Baudin
				T3NN		10.85	35.90	Baudin
Agronomic traits	PH	*Qph.sau-3H*	3H	T2LN	*bpb3433483*–*bpb3257096*	22.62	59.40	CN4027
				T3LN		17.73	50.30	CN4027
				T2NN		20.11	53.80	CN4027
				T3NN		24.45	62.20	CN4027
		*Qph.sau-5H*	5H	T3LN	*bpb3255731*–*bpb3270337*	3.69	13.70	CN4027
		*Qph.sau-7H*	7H	T3NN	*bpb4186071*–*bpb3276000*	3.95	14.50	CN4027
	SL	*Qsl.sau-1H*	1H	T3NN	*bpb3264968*–*bpb3265196*	3.24	12.10	CN4027
		*Qsl.sau-2H*	2H	T2LN	*bpb6272661*–*bpb4595330*	4.60	16.30	CN4027
				T2NN		4.84	17.00	CN4027
		*Qsl.sau-3H.1*	3H	T2LN	*bpb3263863*–*bpb3912056*	4.75	16.80	Baudin
		*Qsl.sau-3H.2*	3H	T2LN	*bpb3263664*–*bpb3987445*	3.31	12.50	Baudin
		*Qsl.sau-6H*	6H	T3LN	*bpb3270443*–*bpb4185288*	3.27	12.10	Baudin
	AL	*Qal.sau-3H*	3H	T2LN	*bpb3664665*–*bpb3264570*	9.19	30.10	CN4027
				T3LN		3.30	12.30	CN4027
				T2NN		5.91	20.30	CN4027
				T3NN		7.96	26.80	CN4027
		*Qal.sau-7H*	7H	T3LN	*bpb4190923*–*bpb3274113*	3.11	11.50	Baudin
				T3NN		3.59	13.10	Baudin
	LDR	*Qldr.sau-3H*	3H	T2LN	*bpb4169758*–*bpb3257096*	6.28	21.60	CN4027
				T3LN		11.27	35.80	CN4027
				T2NN		8.24	27.30	CN4027
				T3NN		9.78	32.00	CN4027
	GP	*Qgp.sau-1H.1*	1H	T3LN	*bpb3265422*–*bpb3267669*	3.52	12.90	CN4027
		*Qgp.sau-1H.2*	1H	T3LN	*bpb3662472*–*bpb3930319*	3.83	14.00	CN4027
		*Qgp.sau-2H*	2H	T2NN	*bpb3986375*–*bpb4011397*	3.94	14.00	Baudin
		*Qgp.sau-3H*	3H	T3LN	*bpb3433483*–*bpb6283337*	3.92	14.30	Baudin
				T2NN		4.84	17.00	Baudin
				T3NN		7.21	24.70	Baudin
		*Qgp.sau-4H*	4H	T3LN	*bpb3264616*–*bpb3257770*	3.21	11.90	CN4027

LN, low nitrogen; NN, normal nitrogen; LOD, logarithm of odds; PVE, phenotype variance explained; RSA, root–shoot ratio; SDW, shoot dry matter weight; TDW, total dry matter weight; PH, plant height; ARD, average root diameter; LRSA, lateral root surface area; LRV, lateral root volume; ARTN, adventitious root tip number; LRLP, lateral root length percentage; ARLP, adventitious root length percentage; ARSAP, adventitious root surface area percentage; LRVP, lateral root volume percentage; ARVP, adventitious root volume percentage; SN, spike number per plant; GN, grain number per spike; TGW, 1000-grain weight; GY, grain yield; StDW, straw dry weight per plant; ADW, aboveground dry weight per plant; HI, harvest index; PH, plant height; SL, spike length; LDR, lodging resistance; GP, growth period; T, trial.

## Data Availability

All data generated or analyzed during this study are included in this published article and its supplementary information files.

## Supplementary Materials

The supporting information can be downloaded at: https://www.mdpi.com/article/10.3390/ijms24108736/s1.
