# Supplementary material for: QTL Mapping of Agronomic and Physiological Traits at the Seedling and Maturity Stages under Different Nitrogen Treatments in Barley
Source: Int J Mol Sci. 2023 May 13;24(10):8736. doi: 10.3390/ijms24108736 (PMC10217864; doi:10.3390/ijms24108736)
Supplement: Supplementary file 1 [file ijms-24-08736-s001.zip › ijms-2367611-supplementary.pdf]

Supplementary Material

Table S1. Nutrient solution ingredients for barely seedling growth.

Ingredient	Concentration(mmol·L^{-1})	Ingredient	Concentration(mmol·L^{-1})
K_2SO_4	0.75	$MnSO_4 \cdot H_2O$	1×10^{-3}
KH_2PO_4	0.2	$ZnSO_4 \cdot 7H_2O$	1×10^{-3}
$MgSO_4 \cdot 7H_2O$	0.65	$CuSO_4 \cdot 5H_2O$	0.5×10^{-3}
EDTA-Fe	0.1	$(NH_4)_6Mo_7O_{24} \cdot 4H_2O$	0.05×10^{-3}
H_3BO_4	1×10^{-3}	$Ca(NO_3)_2 \cdot 4H_2O$	2.0/0.5

Table S2. Basic physiochemical properties of soil before planting.

Index	pH	OM(g·kg-1)	TN(g·kg-1)	AN(mg·kg-1)	AP(mg·kg-1)	AK(mg·kg-1)
2017	5.5	16.9	1.25	53.6	13.6	81.8
2018	5.4	17.7	1.38	54.8	14.5	83.2

Table S3. Quantitative trait locus (QTL) clusters in barley.

Codes	Chr	Marker intervals	No. of QTL	QTL
C1	2H	*bpb3911226-bpb4595330*	3	*Qgy.sau-2H, Qhi.sau-2H.1, Qsl.sau-2H,*
C2	3H	*bpb3271241-bpb3917957*	6	*Qlrsap.sau-3H, Qarsap.sau-3H, Qlrlp.sau-3H, Qarlp.sau-3H, Qlrvp.sau-3H, Qarvp.sau-3H*
C3	3H	*bpb6282426-bpb3264570*	7	*Qsn.sau-3H, Qgn.sau-3H, Qgy.sau-3H, Qadw.sau-3H.1, Qhi.sau-3H, Qsl.sau-3H.1, Qal.sau-3H*
C4	3H	*bpb3433483-bpb3257096*	10	*Qtgw.sau-3H, Qstdw.sau-3H, Qadw.sau-3H.2, Qph.sau-3H, Qldr.sau-3H, Qgp.sau-3H, Qsl.sau-3H, Qsdw.sau-3H, Qph.sau-3H, Qtdw.sau-3H*
C5	6H	*bpb3266659-bpb3910909*	3	*Qlrsa.sau-6H, Qlrv.sau-6H, Qartn.sau-6H*
C6	6H	*bpb3665654-bpb5256842*	4	*Qlrlp.sau-6H.1, Qlrsap.sau-6H, Qarsap.sau-6H, Qarlp.sau-6H.1*
C7	6H	*bpb3271375-bpb3257775*	3	*Qlrlp.sau-6H.2, Qarlp.sau-6H.2, Qtgw.sau-6H*

Table S4. Analysis of candidate genes for stable QTL.

Stable QTL	Putative candidate gene ID	Gene annotation
Qgn.sau-3H	*HORVU3Hr1G063180.1*	glutamate dehydrogenase 2
Qgy.sau-3H	*HORVU3Hr1G063050.8*	glutamate synthase 2
Qhi.sau-3H	*HORVU3Hr1G062150.1*	NAC domain containing protein 2
	HORVU3Hr1G061560.1	NAC domain containing protein 73
	HORVU3Hr1G061710.1	L-asparaginase
	HORVU3Hr1G066050.1	Transcription factor bHLH62
	HORVU3Hr1G062240.1	Transcription factor bHLH85
	HORVU3Hr1G066600.1	Transcription factor bHLH87
Qph.sau-3H	*HORVU3Hr1G088800.2*	NAC domain containing protein 2
Qldr.sau-3H	*HORVU3Hr1G091720.2*	NAC domain containing protein 3
Qgp.sau-3H	*HORVU3Hr1G094170.1*	NAC domain containing protein 32
	HORVU3Hr1G090440.4	NAC domain containing protein 35
	HORVU3Hr1G083330.1	NAC domain containing protein 75
	HORVU3Hr1G083820.9	NAC domain protein
	HORVU3Hr1G089410.1	NAC domain protein
	HORVU3Hr1G090920.1	NAC domain protein
	HORVU3Hr1G095880.1	NAC domain protein
	HORVU3Hr1G089840.1	L-asparaginase
	HORVU3Hr1G096430.2	Transcription factor bHLH35

	HORVU3Hr1G093310.2	Transcription factor bHLH62
Qtgw.sau-2H	*HORVU2Hr1G080990.1*	seed storage 2S albumin superfamily protein
